# Complete genome sequence of *Thermosphaera aggregans* type strain (M11TL^T^)

**DOI:** 10.4056/sigs.821804

**Published:** 2010-06-15

**Authors:** Stefan Spring, Reinhard Rachel, Alla Lapidus, Karen Davenport, Hope Tice, Alex Copeland, Jan-Fang Cheng, Susan Lucas, Feng Chen, Matt Nolan, David Bruce, Lynne Goodwin, Sam Pitluck, Natalia Ivanova, Konstantinos Mavromatis, Galina Ovchinnikova, Amrita Pati, Amy Chen, Krishna Palaniappan, Miriam Land, Loren Hauser, Yun-Juan Chang, Cynthia C. Jeffries, Thomas Brettin, John C. Detter, Roxanne Tapia, Cliff Han, Thomas Heimerl, Fabian Weikl, Evelyne Brambilla, Markus Göker, James Bristow, Jonathan A. Eisen, Victor Markowitz, Philip Hugenholtz, Nikos C Kyrpides, Hans-Peter Klenk

**Affiliations:** 1 DSMZ - German Collection of Microorganisms and Cell Cultures GmbH, Braunschweig, Germany; 2 University of Regensburg, Archaeenzentrum, Regensburg, Germany; 3 DOE Joint Genome Institute, Walnut Creek, California, USA; 4 Los Alamos National Laboratory, Bioscience Division, Los Alamos, New Mexico, USA; 5 Lawrence Livermore National Laboratory, Livermore, California, USA; 6 Oak Ridge National Laboratory, Oak Ridge, Tennessee, USA; 7 University of California Davis Genome Center, Davis, California, USA; 8 Biological Data Management and Technology Center, Lawrence Berkeley National Laboratory, Berkeley, California, USA

**Keywords:** hyperthermophile, strictly fermentative metabolism, sulfur reduction, obligate anaerobic, hot solfataric spring, *Desulfurococcaceae*, *Crenarchaeota*, GEBA

## Abstract

*Thermosphaera aggregans* Huber *et al*. 1998 is the type species of the genus *Thermosphaera*, which comprises at the time of writing only one species. This species represents archaea with a hyperthermophilic, heterotrophic, strictly anaerobic and fermentative phenotype. The type strain M11TL^T^ was isolated from a water-sediment sample of a hot terrestrial spring (Obsidian Pool, Yellowstone National Park, Wyoming). Here we describe the features of this organism, together with the complete genome sequence and annotation. The 1,316,595 bp long single replicon genome with its 1,410 protein-coding and 47 RNA genes is a part of the *** G****enomic **** E****ncyclopedia of **** B****acteria and **** A****rchaea * project.

## Introduction

Strain M11TL^T^ (= DSM 11486) is the type strain of the species *Thermosphaera aggregans* [1]. M11TL^T^ is the only strain of this species available from a culture collection and was isolated from water and sediment samples of a terrestrial circumneutral hot solfataric spring (“Obsidian Pool”) located in the Mud Volcano area of the Yellowstone National Park, Wyoming. For the isolation of this strain from enrichment cultures a then (1988) novel approach was used. Single cells with a distinct morphotype were directly selected for cultivation by a newly developed micromanipulation technique consisting of a modified inverse microscope equipped with a strongly focused infrared laser (“optical tweezers”) [2].

No other cultivated strain belonging to the species *T. aggregans* has been described. The closest related type strain of a species with a sequenced 16S rRNA gene, *Desulfurococcus mobilis* [3], shows 4.5% sequence difference. Uncultured representatives of the *Desulfurococceae* with a high degree of 16S rRNA sequence similarity (>99.7%) to strain M11TL^T^ were identified in two other circumneutral terrestrial hot springs in the United States [4,5], whereas no sequences of closely related archaea could be retrieved from high temperature acidic or marine environments using cultivation-independent approaches. Consequently, it appears that cells of this species are restricted to hot, pH neutral, terrestrial springs.

The complete genome sequences of the related species *Desulfurococcus kamchatkensis* strain 1221n^T^ [6] and *Staphylothermus marinus *strain F1T [7] were recently finished, so that three genomes of closely related hyperthermophilic, organotrophic and neutrophilic *Crenarchaeota* are available for a detailed comparison. This is especially interesting for an understanding of the genetic basis of sulfur respiration in this clade, because, albeit, all three species are capable to produce H_2_S, the benefit of sulfur reduction varies drastically. Here we present a summary classification and a set of features for *T. aggregans* strain M11TL^T^, together with the description of the complete genomic sequencing and annotation.

## Classification and features

In reconstructed phylogenetic trees *T. aggregans* and representatives of the genera *Sulfophobococcus*, *Desulfurococcus* and *Staphylothermus* form a relatively stable distinct branch within the family *Desulfurococcaceae*, order *Desulfurococcales*. Most members of this clade thrive in terrestrial habitats and are characterized by having a coccoid morphology and a strictly anaerobic, heterotrophic metabolism.

**Figure 1:**
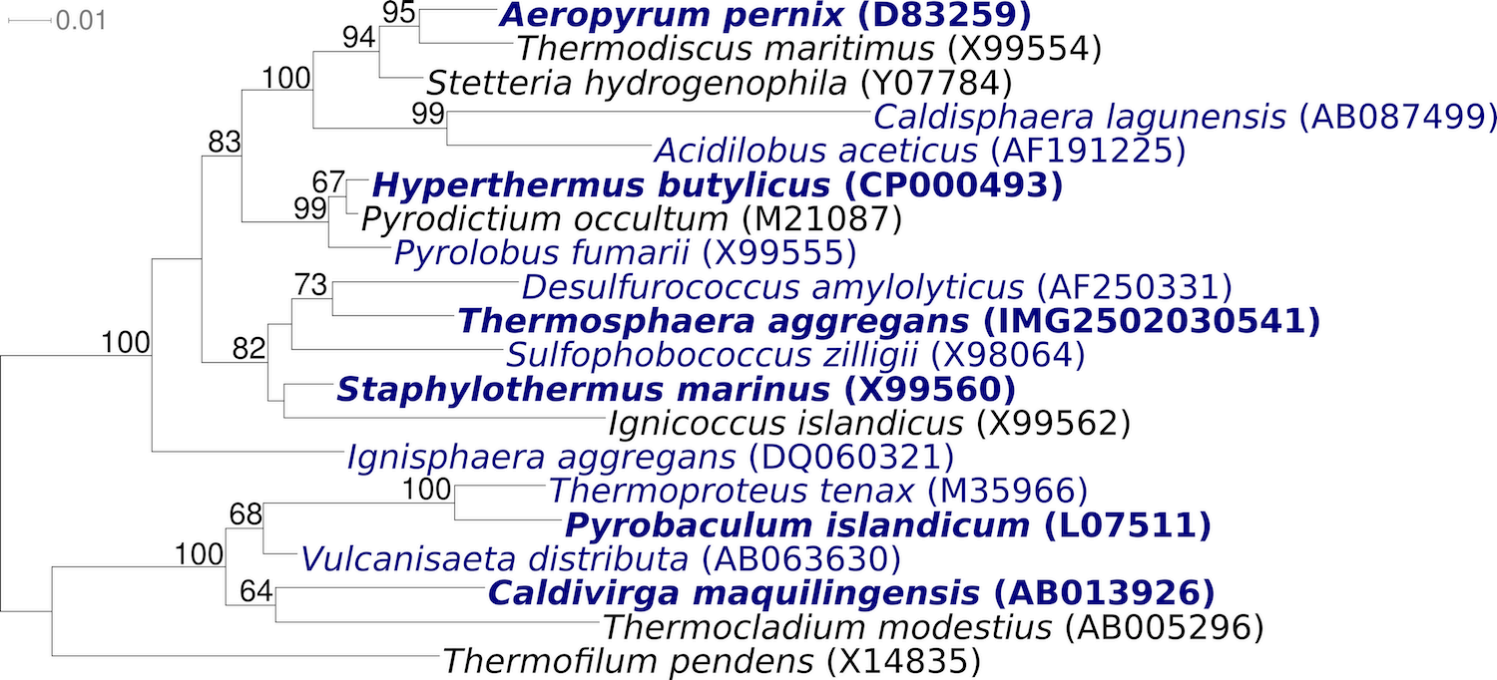
Phylogenetic tree highlighting the position of *T. aggregans* relative to the other type strains of the other genera within the family *Desulfurococcaceae*. The tree was inferred from 1,307 aligned characters [8,9] of the 16S rRNA gene sequence under the maximum likelihood criterion [10] and rooted in accordance with the current taxonomy. The branches are scaled in terms of the expected number of substitutions per site. Numbers above branches are support values from 200 bootstrap replicates [11] if larger than 60%. Lineages with type strain genome sequencing projects registered in GOLD [12] are shown in blue, published genomes in bold.

Figure 1 shows the phylogenetic neighborhood of *T. aggregans* strain M11TL^T^ in a 16S rRNA based tree. The genome of strain M11TL^T^ contains only a single 16S rRNA gene that differs by one nucleotide from the previously published 16S rRNA gene sequence generated from the same strain (X99556), which contains nine ambiguous base calls. The difference between the genome data and the here reported 16S rRNA gene sequence is most likely due to sequencing errors in the previously reported sequence (NAS).

**Figure 2:**
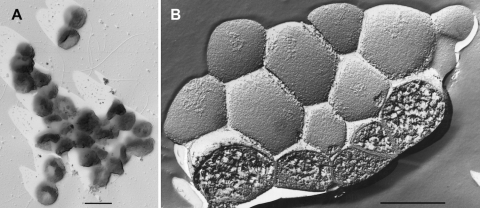
Transmission electron micrographs of cells of *T. aggregans* strain M11TL^T^. A: cells were shadowed with platinum; B: cells were freeze-etched. Scale bars, 1 µm

Cells of *T. aggregans* M11TL^T^ are regular cocci that preferentially grow in grape-like aggregates consisting of five to several hundred individuals [1]. They have normally dimensions between 0.2 and 0.8 µm (Figure 2 and Table 1), but under suboptimal growth conditions a swelling of cells was observed leading to dimensions of up to 3.5 µm. Flagella-like appendages are formed but motility was not described [1]. The cell envelope consists of a cytoplasmic membrane that is covered by an amorphous layer of unknown composition. A regularly arrayed surface-layer protein was not detected by transmission electron microscopy of freeze-etched specimen, i.e. under experimental conditions which allow instant visualization of S-layers in cells of related genera.

**Table 1:** Classification and general features of *T. aggregans* strain M11TL^T^ according to the MIGS recommendations [13]

**MIGS ID**	**Property**	**Term**	**Evidence code**
	Current classification	Domain *Archaea*	TAS [14]
		Phylum *Crenarchaeota*	TAS [15]
		Class *Thermoprotei*	TAS [16]
		Order *Desulfurococcales*	TAS [17]
		Family *Desulfurococcaceae*	TAS [3]
		Genus *Thermosphaera*	TAS [1]
		Species *Thermosphaera aggregans*	TAS [1]
		Type strain M11TL	TAS [1]
	Gram stain	not reported	
	Cell shape	coccoid, grapelike aggregates	TAS [1]
	Motility	not reported (flagella present)	TAS [1]
	Sporulation	non-sporulating	TAS [1]
	Temperature range	67-90°C	TAS [1]
	Optimum temperature	85°C	TAS [1]
	Salinity	not determined	
MIGS-22	Oxygen requirement	aerobic	TAS [1]
	Carbon source	yeast extract, peptone, gelatin, amino acids, heat-treated xylan, glucose	TAS [1]
	Energy source	see above	TAS [1]
MIGS-6	Habitat	hot, pH neutral, solfataric springs	TAS [1]
MIGS-15	Biotic relationship	unknown	
MIGS-14	Pathogenicity	none	TAS [18]
	Biosafety level	1	TAS [18]
	Isolation	water/sediment sample	TAS [1]
MIGS-4	Geographic location	Obsidian Pool, Yellowstone National Park, Wyoming USA	TAS [1]
MIGS-5	Sample collection time	1994 or before	NAS
MIGS-4.1 MIGS-4.2	Latitude Longitude	44.806 -110.448	NAS NAS
MIGS-4.3	Depth	not reported	
MIGS-4.4	Altitude	not reported	

Evidence codes - IDA: Inferred from Direct Assay (first time in publication); TAS: Traceable Author Statement (i.e., a direct report exists in the literature); NAS: Non-traceable Author Statement (i.e., not directly observed for the living, isolated sample, but based on a generally accepted property for the species, or anecdotal evidence). These evidence codes are from the Gene Ontology project [19]. If the evidence code is IDA, then the property was directly observed for a live isolate by one of the authors or an expert mentioned in the acknowledgments.

Strain M11TL^T^ is hyperthermophilic and grows optimally at 85°C, the temperature range for growth is 67 to 90°C. The pH range for growth is 5.0 - 7.0 with an optimum at pH 6.5. The strain grows optimally in the absence of exogenous NaCl, but can be adapted to salt concentrations of up to 0.7%. The doubling time under optimal growth conditions is 110 min [1].

*T. aggregans* M11TL^T^ is strictly anaerobic and grows heterotrophically on yeast extract, peptone, gelatin, amino acids, heat-treated xylan, and glucose. Upon growth on yeast extract and peptone, the fermentation products acetate, isovalerate, CO_2_ and H_2_ were identified. No growth on meat extract, amylose, glycogen, cellulose, cellobiose, maltose, raffinose, pyruvate and acetate was found. Growth of strain M11TL^T^ is inhibited by sulfur and H_2_ [1]. It has been reported that addition of sulfur (0.05% w/v) to growing cultures leads to complete inhibition of growth, production of H_2_S and finally lysis of cells. A growth-inhibiting effect of sulfur was also reported for *Sulfophobococcus zilligii* [20], but is absent in the closely related genera *Desulfurococcus* and *Staphylothermus*. In contrast, in both of the latter genera sulfur has either a stimulatory effect [21] or is even required for growth [22] and reduced to H_2_S. Interestingly, an inhibiting effect in cultures of *T. aggregans* and *S. zilligii* was not observed, if growth media were supplemented with the sulfur compounds sulfide, sulfite or thiosulfate [1,20], so that this effect seems to be restricted to elemental sulfur. The inhibiting effect of H_2_ on growth is reversible and can be explained by a product inhibition of sensitive hydrogenases, which may be required for the disposal of reducing equivalents as hydrogen during fermentation.

### Chemotaxonomy

The lipid composition of *T. aggregans* was analyzed by thin-layer chromatography. Core lipids were mainly composed of acyclic and cyclic dibiphytanyl glycerol tetraethers with one to four pentacyclic rings. In addition, traces of diphytanyl glycerol diethers were also detected [1]. The presence of cyclic tetraether lipids in this species seems to be a diagnostic trait, because thus far these lipids were not detected in the related genera *Sulfophobococcus* [20], *Staphylothermus* [22,23] or *Desulfurococcus* [24]. Unfortunately, no data about the polyamine, quinone or cytochrome composition in *T. aggregans* are currently available. However, respiratory lipoquinones could not be detected in *Sulfophobococcus zilligii*, *Desulfurococcus mucosus* and *Desulfurococcus mobilis* [20,25], whereas homospermidine was identified as principal polyamine in several species closely related to *T. aggregans* [26].

## Genome sequencing and annotation

### Genome project history

This organism was selected for sequencing on the basis of its phylogenetic position, and is part of the *** G****enomic* *** E****ncyclopedia of* *** B****acteria and* *** A****rchaea * project [27]. The genome project is deposited in the Genomes OnLine Database [12] and the complete genome sequence in GenBank. Sequencing, finishing and annotation were performed by the DOE Joint Genome Institute (JGI). A summary of the project information is shown in Table 2.

**Table 2:** Genome sequencing project information

**MIGS ID**	**Property**	**Term**
MIGS-31	Finishing quality	Finished
MIGS-28	Libraries used	Two 454 pyrosequence libraries, standard and pairs end (8 kb insert size) and one Illumina library (300 bp insert size)
MIGS-29	Sequencing platforms	454 Titanium, Illumina GAii
MIGS-31.2	Sequencing coverage	104.8× 454 pyrosequence, 277× Illumina
MIGS-30	Assemblers	Newbler, Velvet, phrap
MIGS-32	Gene calling method	Prodigal, GenePRIMP
	GenBank ID	CP001939
	GenBank Date of Release	May 17, 2010
	GOLD ID	Gi02946
	NCBI project ID	36571
	Database: IMG-GEBA	2501939626
MIGS-13	Source material identifier	DSM 11486
	Project relevance	GEBA

### Growth conditions and DNA isolation

*T. aggregans* M11TL^T^, DSM 11486, was grown anaerobically in DSMZ medium 817 [28] at 85°C. DNA was isolated from 1-1.5 g of cell paste using Jetflex Genomic DNA Purification kit (GENOMED 600100) according to the manufacturers instructions.

### Genome sequencing and assembly

The genome of *Thermosphaera aggregans* DSM 11486 was sequenced using a combination of Illumina [29] and 454 [30] technologies. An Illumina GAii shotgun library with total reads of 360 Mb, a 454 Titanium draft library with average read length of 327 bases, and a paired end 454 library with average insert size of 8.2 Kb were generated for this genome. All general aspects of library construction and sequencing can be found at http://www.jgi.doe.gov/. Illumina sequencing data were assembled with VELVET [31], and the consensus sequences were shredded into 1.5 kb overlapped fake reads and assembled together with the 454 data. Draft assemblies were based on 136.2 Mbp Mb 454 data. Newbler parameters are -consed -a 50 -l 350 -g -m -ml 20. The initial assembly contained two contigs in one scaffold. We converted the initial 454 assembly into a phrap assembly by making fake reads from the consensus, collecting the read pairs in the 454 paired end library. The Phred/Phrap/Consed software package (www.phrap.com) was used for sequence assembly and quality assessment in the following finishing process. After the shotgun stage, reads were assembled with parallel phrap. Possible mis-assemblies were corrected with gapResolution (unpublished; http://www.jgi.doe.gov/), Dupfinisher, or sequencing cloned bridging PCR fragments with subcloning or transposon bombing [32]. Gaps between contigs were closed by editing in Consed, by PCR and by Bubble PCR primer walks (J-F. Chan, unpublished). No additional reactions were necessary to close gaps. Illumina reads were used to improve the final consensus quality using an in-house developed tool (the Polisher). The completed genome sequence has an error rate of less than 1 in 100,000 bp.

### Genome annotation

Genes were identified using Prodigal [33] as part of the Oak Ridge National Laboratory genome annotation pipeline, followed by a round of manual curation using the JGI GenePRIMP pipeline [34]. The predicted CDSs were translated and used to search the National Center for Biotechnology Information (NCBI) nonredundant database, UniProt, TIGRFam, Pfam, PRIAM, KEGG, COG, and InterPro databases. Additional gene prediction analysis and manual functional annotation was performed within the Integrated Microbial Genomes Expert Review (IMG-ER) platform [35].

## Genome properties

**Figure 3:**
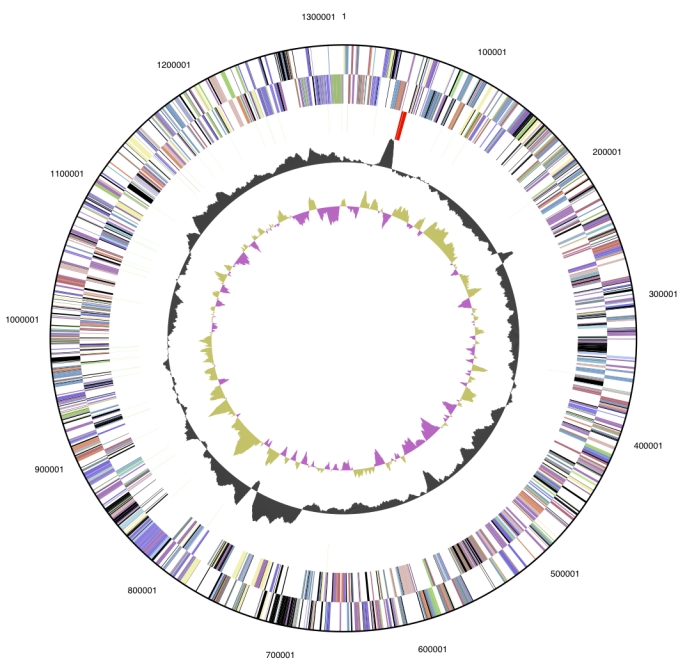
Graphical circular map of the genome. From outside to the center: Genes on forward strand (color by COG categories), Genes on reverse strand (color by COG categories), RNA genes (tRNAs green, rRNAs red, other RNAs black), GC content, GC skew.

The genome consists of a 1,316,595 bp long chromosome with a 46.7% GC content (Table 3 and Figure 3). Of the 1,457 genes predicted, 1,419 were protein-coding genes, and 47 RNAs; 23 pseudogenes were identified. The majority of the protein-coding genes (62.7%) were assigned with a putative function while those remaining were annotated as hypothetical proteins. The distribution of genes into COGs functional categories is presented in Table 4.

**Table 3:** Genome Statistics

**Attribute**	**Value**	**% of Total**
Genome size (bp)	1,316,595	100.00%
DNA coding region (bp)	1,221,613	92.79%
DNA G+C content (bp)	615,302	46.73%
Number of replicons	1	
Extrachromosomal elements	0	
Total genes	1,457	100.00%
RNA genes	47	3.23%
rRNA operons	1	
Protein-coding genes	1,419	96.77%
Pseudo genes	23	1.58%
Genes with function prediction	914	62.73%
Genes in paralog clusters	79	5.42%
Genes assigned to COGs	990	67.95%
Genes assigned Pfam domains	1,007	69.11%
Genes with signal peptides	131	8.99%
Genes with transmembrane helices	323	22.17%
CRISPR repeats	1	

**Table 4:** Number of genes associated with the general COG functional categories

**Code**	**value**	**%age**	**Description**
J	152	10.8	Translation, ribosomal structure and biogenesis
A	2	0.1	RNA processing and modification
K	50	3.5	Transcription
L	58	4.1	Replication, recombination and repair
B	2	0.1	Chromatin structure and dynamics
D	0	0.0	Cell cycle control, cell division, chromosome partitioning
Y	0	0.0	Nuclear structure
V	12	0.9	Defense mechanisms
T	17	1.2	Signal transduction mechanisms
M	35	2.5	Cell wall/membrane biogenesis
N	9	0.6	Cell motility
Z	0	0.0	Cytoskeleton
W	0	0.0	Extracellular structures
U	13	0.9	Intracellular trafficking, secretion, and vesicular transport
O	44	3.1	Posttranslational modification, protein turnover, chaperones
C	96	6.8	Energy production and conversion
G	55	3.9	Carbohydrate transport and metabolism
E	71	5.0	Amino acid transport and metabolism
F	32	2.3	Nucleotide transport and metabolism
H	49	3.5	Coenzyme transport and metabolism
I	14	1.0	Lipid transport and metabolism
P	70	5.0	Inorganic ion transport and metabolism
Q	4	0.3	Secondary metabolites biosynthesis, transport and catabolism
R	176	12.5	General function prediction only
S	467	33.1	Function unknown
-	467	33.1	Not in COGs

## Insights from the genome sequence

### Substrate uptake and hydrolysis

*T. aggregans* grows optimally in complex media and uses peptides and carbohydrates as principal carbon and energy sources, which have to be transported inside the cell. Complex extracellular substrates that cannot be transported could be attacked by membrane bound enzymes like a subtilisin-like serine protease (Tagg_1197) or a putative pullulanase (Tagg_1302). Several genes of supposed transporters of the ABC type were identified in the annotated genome, which catalyze the energy-dependent uptake of carbohydrates (Tagg_0544-0547; Tagg_1122-1125), nucleosides (Tagg_0246-0249; Tagg_1129-1132) or peptides (Tagg_0288-0291; Tagg_0952-0955; Tagg_1113-1117). Amino acids and other small molecules are probably transported across the cytoplasmic membrane by various secondary transporters belonging to the sodium:solute symporter family (Tagg_0251, Tagg_0258), the sodium:neurotransmitter symporter family (Tagg_0418) and the sodium:dicarboxylate symporter family (Tagg_0524). The sodium-motive force required for the uptake of small solutes is possibly generated by sodium ion-proton antiporters (e.g., Tagg_0296), whereas no genes encoding any of the known sodium ion-translocating decarboxylases could be identified.

Within the cell oligopeptides are degraded by several distinct peptidases, represented by Tagg_0523 (trypsin-like serine protease), Tagg_0073 (aminopeptidase), Tagg_1142 (Xaa-Pro aminopeptidase), Tagg_0908 (zinc-dependent peptidase), Tagg_0282 (thermophilic metallo-aminopeptidase), and Tagg_0456 (thermostable zinc-dependent carboxypeptidase). On the other hand, glycosidases might be involved in the degradation of complex oligosaccharides. Three different types of glycoside hydrolases were identified, which belong to family 1 (Tagg_1110), family 4 (Tagg_1191) and family 57 (Tagg_0640). A beta-glycosidase represented by the gene locus Tagg_1110 has been already identified before the here reported complete genome sequencing and was expressed in *Escherichia coli* as a recombinant protein. A crystal structure of this *T. aggregans* enzyme was determined in order to identify factors which could be responsible for its thermostability [36,37].

### Catabolism of amino acids

Within the cell free amino acids are probably fermentatively degraded by a pathway that is commonly found in anaerobic hyperthermophilic archaea [6,38]. In a first step amino groups are removed from the carbon skeleton by several distinct aminotransferases, which could be affiliated to class I/II (Tagg_0668), class III (Tagg_0004) and class V (Tagg_1145). The final acceptor of the released amino groups is likely 2-oxoglutarate thereby resulting in the accumulation of glutamate, which is subsequently oxidatively deaminated by the activity of a glutamate dehydrogenase (Tagg_1073). Upon deamination of amino acids the resulting 2-oxoacid derivates can be oxidatively decarboxylated to the respective coenzyme A (CoA) derivates by various 2-oxoacid-ferredoxin oxidoreductases having broad substrate specificity. Genes encoding subunits of all known archaeal 2-oxoacid-ferredoxin oxidoreductases could be identified in the *T. aggregans* genome and represent pyruvate-ferredoxin oxidoreductase (Tagg_0386-0389), 2-oxoglutarate-ferredoxin oxidoreductase (Tagg_0390-0393), 2-oxoisovalerate-ferredoxin oxidoreductase (Tagg_0826-0829) and indolepyruvate-ferredoxin oxidoreductase (Tagg_0224, Tagg_0225). In addition, three different aldehyde-ferredoxin oxidoreductases are encoded in the *T. aggregans* genome, indicating that the 2-oxoacid-ferredoxin oxidoreductases may also catalyze a non oxidative decarboxylation reaction that leads to the corresponding aldehydes as described in *Pyrococcus furiosus* [39]. One of the annotated aldehyde-ferredoxin oxidoreductases (Tagg_0120) is of special interest, because it could be acquired by lateral gene transfer. The most similar homologous proteins identified in a BLAST database search were enzymes of the bacteria *Desulfohalobium retbaense* [40] (Dret_2319, 47% amino acid identity), *Pelotomaculum thermopropionicum* (PTH_2897, 46% identity) and *Desulfonatronospira thiodismutans* (DthioDRAFT_3258, 45% identity). Besides the oxidation of aldehydes to carboxylic acids by aldehyde-ferredoxin oxidoreductases an alternative pathway appears to exist that would be based on the reduction of aldehydes to the corresponding alcohols by alcohol dehydrogenases. Genes of two different types of alcohol dehydrogenases were identified, a zinc binding (Tagg_0918) and an iron containing enzyme (Tagg_0471). The reduction of aldehydes leads to the oxidation of NAD(P)H, whereas the oxidation to carboxylic acids produces reduced ferredoxin, hence, a function of both pathways could be a balancing of the cellular redox state [38].

A gene encoding an arginine decarboxylase belonging to COG1166, which is rarely found among *Archaea*, was detected in the genome of *Staphylothermus marinus* [7] and could be also identified in the genomes of *T. aggregans* (Tagg_0502; speA in *Escherichia coli*) and *Desulfurococcus kamchatkensis* [41]. It is likely that this enzyme does not participate in the degradation of amino acids, but is part of a biosynthetic pathway leading to the polyamine spermidine. This is supported by the identification of genes for an agmatinase (Tagg_1172; speB) and a spermidine synthase (Tagg_0403; speE), which could be involved in the synthesis of spermidine along with the arginine decarboxylase.

### Catabolism of monosaccharides

In *T. aggregans* sugars can be oxidized to pyruvate via a modified glycolytic Embden-Meyerhof-Parnas pathway as described for *Pyrococcus furiosus* and several other hyperthermophilic archaea [42]. However, in difference to *Pyrococcus furiosus*, which uses ADP-dependent enzymes for the phosphorylation of glucose (ADP-GLK) and fructose-6-phosphate (ADP-PFK), in *T. aggregans* corresponding ATP-dependent kinases (Tagg_0486 and Tagg_0553, respectively) are involved in the first steps of glycolysis. The key enzyme of the modified Embden-Meyerhof-Parnas pathway in *Archaea* is glyceraldehyde-3-phosphate-ferredoxin oxidoreductase [43] (Tagg_0452), which oxidizes glyceraldehyde-3-phosphate directly to 3-phosphoglycerate without generating ATP using ferredoxin as electron acceptor. The reaction catalyzed by this enzyme seems to be irreversible and two different enzymes designated 3-phosphoglycerate kinase (Tagg_0302) and glyceraldehyde-3-phosphate dehydrogenase (Tagg_0301) are required to synthesize glyceraldehyde-3-phosphate for gluconeogenesis via 2,3-bisphosphoglycerate. The pyruvate generated by glycolysis is further oxidized to acetyl-CoA by a pyruvate-ferredoxin oxidoreductase.

### Energy metabolism

According to the obtained genome data two alternative pathways for synthesizing of ATP can be proposed for *T. aggregans*: ATP could be either gained by substrate-level phosphorylation or by an ATP synthase complex (Tagg_0078-0087) that utilizes a chemiosmotic gradient.

Pyruvate kinase (Tagg_1237) converting phosphoenolpyruvate into pyruvate is presumably used in *T. aggregans* for the regeneration of ATP that is consumed for the activation of hexoses during glycolysis. However, the principal enzymes responsible for substrate-level phosphorylation in hyperthermophilic heterotrophic *Archaea* are mainly ADP-forming acyl-CoA synthetases. It is thought that in *Archaea* these enzymes catalyze primarily the reverse reaction, which leads to the release of a carboxylic acid and coenzyme A, accompanied by the generation of ATP [44]. A succinyl-CoA synthetase (Tagg_1018, Tagg_1019) and two putative acetyl-CoA synthetases were annotated in the *T. aggregans* genome. Subunits of one acetyl-CoA synthetase are encoded on different sites of the genome (Tagg_0340, Tagg_0726), whereas both genes of the other enzyme are located adjacently (Tagg_142, Tagg_143).

In contrast to substrate-level phosphorylation that occurs in the cytoplasm specific membrane-bound complexes are required to establish an electrochemical gradient across the cytoplasmic membrane that can be utilized for ATP production. No heme or lipoquinone synthesis pathways were identified in the annotated genome, thus neither cytochromes nor quinones are probably involved in electron transport pathways leading to an electrochemical potential difference across the cytoplasmic membrane. However, it is possible that a chemiosmotic gradient is generated by the terminal oxidation of reduced ferredoxins at multimeric membrane-bound complexes. At least two distinct gene clusters were identified in the *T. aggregans* genome that could be involved in the oxidation of reduced ferredoxins: Tagg_1025-1036 and Tagg_0624-0636. A third membrane-bound complex is putatively involved in the reoxidation of NADPH (Tagg_0050-0059), but likely not involved in the generation of metabolically useful energy. All of the above mentioned multienzyme complexes are related to an energy-coupling membrane-bound hydrogenase previously identified in *Pyrococcus furiosus* [45]. The structure and possible functions of these complexes are analyzed in detail below.

### MBH-related energy-coupling hydrogenase

Based on similarity with genes of the characterized membrane-bound hydrogenase (MBH) of *Pyrococcus furiosus* it is proposed that the cluster of *T. aggregans* genes located at Tagg_0624-636 represents an enzyme with similar function. Genes involved in the maturation of [NiFe] hydrogenases (Tagg_0621-0623) are located in close proximity to this gene cluster, which further indicates that this enzyme complex functions as hydrogenase. The first eight genes of the *Pyrococcus furiosus* operon encoding the MBH complex display some similarity with subunits of multimeric cation-proton antiporters [46] and are probably involved in proton or sodium ion translocation across the membrane. The remaining genes are homologous to subunits of [NiFe] hydrogenases or NADH-quinone oxidoreductases (complex I of the respiratory chain). Although both enzymes have now different functions, it was postulated that they share a common evolutionary history [47].

**Figure 4:**
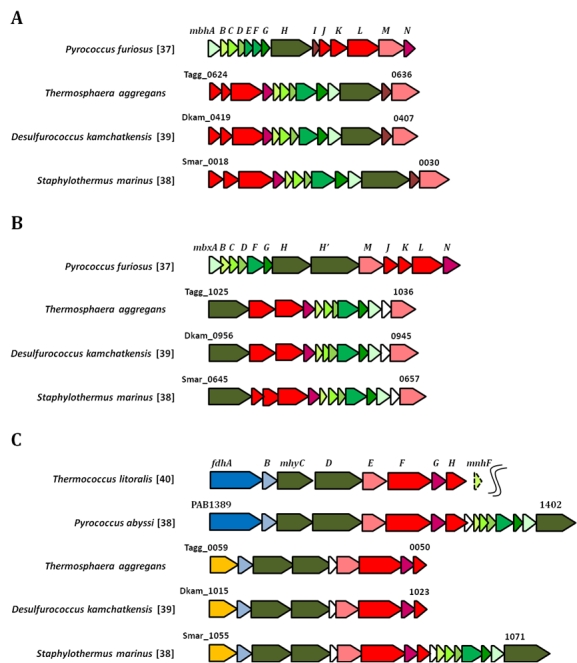
Organization of genes encoding putative membrane-bound multiprotein complexes in *T. aggregans* and related members of *Desulfurococcaceae*. Operons of reference genes with asserted function are shown in the first line of each group. Genes with an assumed homologous function are labeled in the same color. Genes encoding subunits of putative multimeric cation-proton antiporters are shown in shades of green; genes with similarity to subunits of [NiFe] hydrogenases/NADH-quinone oxidoreductases are shown in red colors; genes representing the large and small subunit of supposed formate dehydrogenases are displayed in dark and light blue, respectively; genes labeled in yellow are homologous to genes encoding the alpha-subunit of *Pyrococcus furiosus* sulfide dehydrogenase (sudA); and genes representing hypothetical proteins of unknown function are shown in white. A) MBH-related energy-coupling hydrogenases. B) MBX-related ferredoxin-NADPH oxidoreductases. C) MBX-related ferredoxin-NADPH oxidoreductases. The reference operon of *Thermococcus litoralis* has been retrieved by cloning a fragment of genomic DNA, so that the arrangement of genes following *mnhF* could not be determined.

The enzyme complex of *Pyrococcus furiosus* has been shown to use reduced ferredoxin as electron donor and protons as electron acceptor thereby producing molecular H_2_. In laboratory experiments it could be demonstrated that the production of H_2_ is coupled to proton translocation [31]. The resulting chemiosmotic gradient could then be utilized by a proton-transporting ATP synthase complex. The proposed model of energy coupling by the MBH complex of *Pyrococcus furiosus* has been recently challenged by results of Pisa et al. [35], who found that the ATP synthase complex of *Pyrococcus furiosus* is sodium ion-dependent. A sodium ion-dependence of ATP synthesis would easily explain the presence of sodium ion-proton antiporter genes in close association with hydrogenase genes of the MBH-type in *Pyrococcus furiosus* and representatives of the *Desulfurococcaceae* (Figure 4A). It was postulated that sodium ions would have several advantages compared to protons as coupling ion for growth in anoxic and hot environments [48], so that sodium bioenergetics in *Pyrococcus furiosus* and other hyperthermophilic archaea could reflect an adaptation to the encountered growth conditions.

### MBX-related ferredoxin-NADPH oxidoreductase

In presence of elemental sulfur the ferredoxin-oxidizing, H_2_-evolving MBH complex of *Pyrococcus furiosus* is largely replaced by a homologous membrane-bound complex that is thought to use reduced ferredoxin for the production of NADPH, but does not reduce protons. This complex was designated MBX in *Pyrococcus furiosus* [49] and is also present in sequenced genomes of *Staphylothermus marinus* [7], *Desulfurococcus kamchatkensis* [40] and *T. aggregans* (Tagg_1025-1036). It was postulated that the MBX complex in *Pyrococcus furiosus* supplies NADPH for a coenzyme A-dependent sulfur oxidoreductase. Consequently, an induction of the MBX complex would result in a shift from H_2_ to H_2_S production [49]. Similar to the structure of MBH operons genes encoding multimeric cation-proton antiporters are associated with genes for subunits of [NiFe] hydrogenases/NADH-quinone dehydrogenases (Figure 4B), which may indicate that MBX complexes participate also in the generation of chemiosmotic gradients and electron transport phosphorylation.

In sequenced genomes of *T. aggregans*, *Staphylothermus marinus* and *Desulfurococcus marinus* no genes encoding a cytoplasmic coenzyme A-dependent NADPH sulfur oxidoreductase or other potential cytoplasmic sulfur oxidoreductases were annotated, hence the produced NADPH in this clade of archaea may be utilized by different enzymes.

### Dehydrogenase-linked MBX complex

In the heterotrophic hyperthermophilic archaeon *Thermococcus litoralis* a cluster of genes was identified that resembles known operons of MBH/MBX complexes and is located adjacent to genes coding for a formate dehydrogenase [50]. It was found that *T. litoralis* expresses a formate dehydrogenase that is associated with a membrane-bound [NiFe] hydrogenase of the MBH type resulting in a multimeric enzyme complex which functions as a formate hydrogenlyase cleaving formate into CO_2_ and H_2_. A homologous formate hydrogenlyase operon was identified in the genome of *Pyrococcus abyssi* [51]. It comprises also a conserved set of genes encoding a multimeric sodium ion-proton antiporter, which is probably also present in *T. litoralis*, but could not be detected due to the restricted length of the cloned DNA fragment. Thus, it is likely that in both species the removal of fermentatively produced formate by this enzyme complex is linked to the generation of a chemiosmotic gradient.

Operons encoding related dehydrogenase-linked MBX complexes were also identified in *T. aggregans* (Tagg_0050-0059) and other members of the *Desulfurococcaceae* (Figure 4C). However, the operons found in *T. aggregans*, *Desulfurococcus kamchatkensis* and *Staphylothermus marinus* lack genes coding for the large or *alpha*-subunit of formate dehydrogenase and consequently do not represent formate hydrogenlyases. In place of the *fdhA* gene a gene homologous to the *alpha*-subunit of the sulfide dehydrogenase of *Pyrococcus furiosus* (*sudA*) is present. However, it is now known that this enzyme functions in vivo as reduced ferredoxin-NADP+ oxidoreductase [49]. In general, protein domains or enzyme subunits homologous to *SudA* can occur in various contexts (e.g. as small subunit of glutamate synthase) and transfer electrons from NAD(P)H to an acceptor protein or protein domain [52]. This would suggest that in *T. aggregans* the MBX complex is linked to a NADPH dehydrogenase. Although, at the moment it cannot be deduced what kind of electron acceptor is used by the MBX complex, a reduction of protons or sulfur might be the most reasonable assumption. It is known that some types of [NiFe] hydrogenases can reduce both protons and elemental sulfur [53], so that it could be also possible that the entire complex oxidizes NADPH with either protons or sulfur as electron acceptor depending on the growth conditions. In contrast to *T. aggregans* and *Desulfurococcus kamchatkensis* the operon in *Staphylothermus marinus* comprises genes coding for a multimeric cation-proton antiporter, which could offer an explanation for the different effects of sulfur on the growth response of these species.

According to this hypothesis the coupling of energy metabolism with sulfur reduction was probably present in ancestors of the *Desulfurococcaceae*, but was lost during evolution in *T. aggregans* and *Desulfurococcus kamchatkensis* due to recent gene arrangements. However, this model can still not explain the observed growth-inhibiting effect of sulfur on *T. aggregans*, but not *Desulfurococcus kamchatkensis*.
